# Supplementary material for: Differences in the Accessory Genomes and Methylomes of Strains of Streptococcus equi subsp. equi and of Streptococcus equi subsp. zooepidemicus Obtained from the Respiratory Tract of Horses from Texas
Source: Microbiol Spectr. 2022 Jan 12;10(1):e00764-21. doi: 10.1128/spectrum.00764-21 (PMC8754150; doi:10.1128/spectrum.00764-21)
Supplement: SUPPLEMENTAL FILE 2 — Supplemental material. Download SPECTRUM00764-21_Supp_2_seq17.pdf, PDF file, 0.4 MB [file spectrum00764-21_supp_2_seq17.pdf]

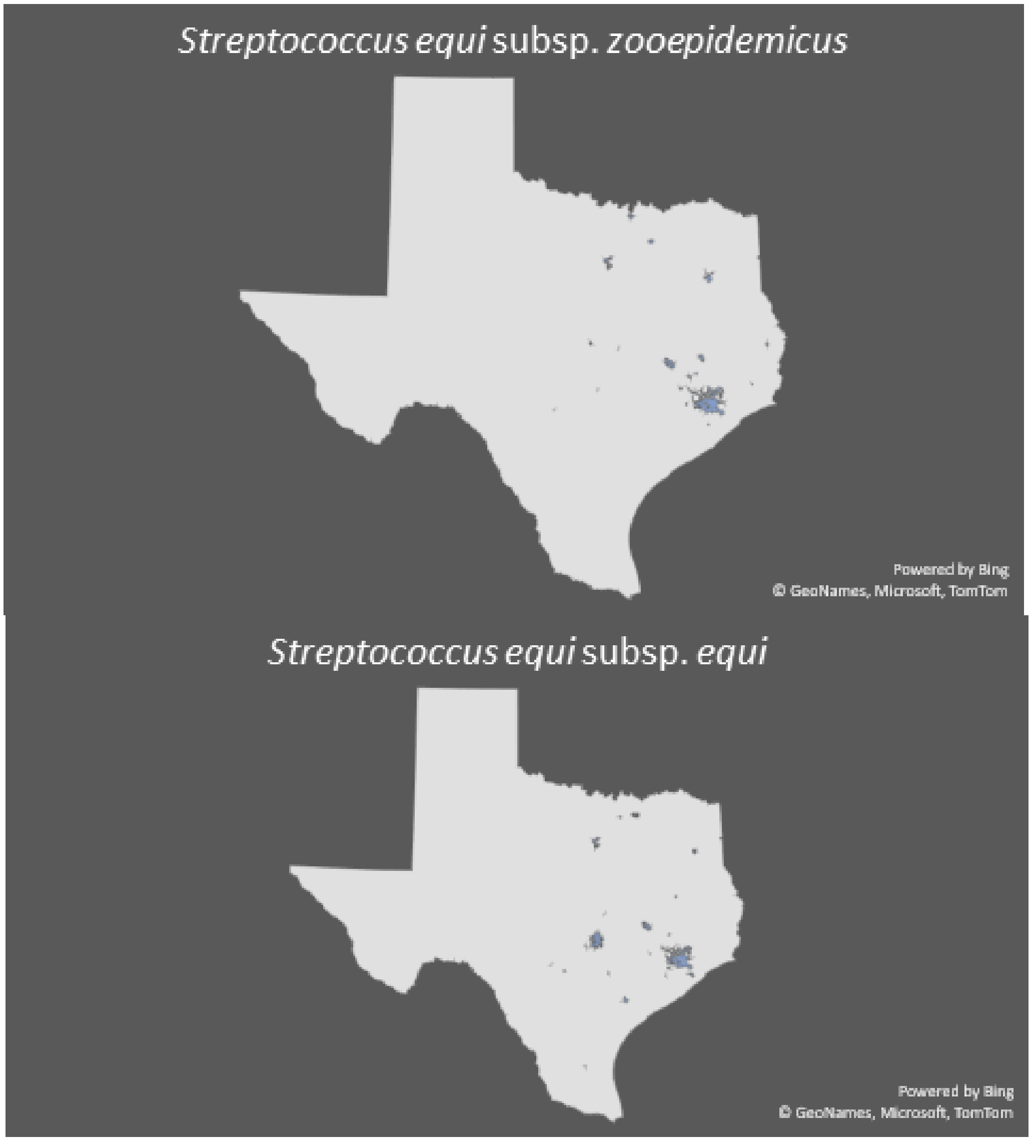

Fig. S1. Geographic distribution of Texas isolates for which origin was known: *Streptococcus equi* subsp. *zooepidemicus* (top) and subsp. *equi* (bottom).

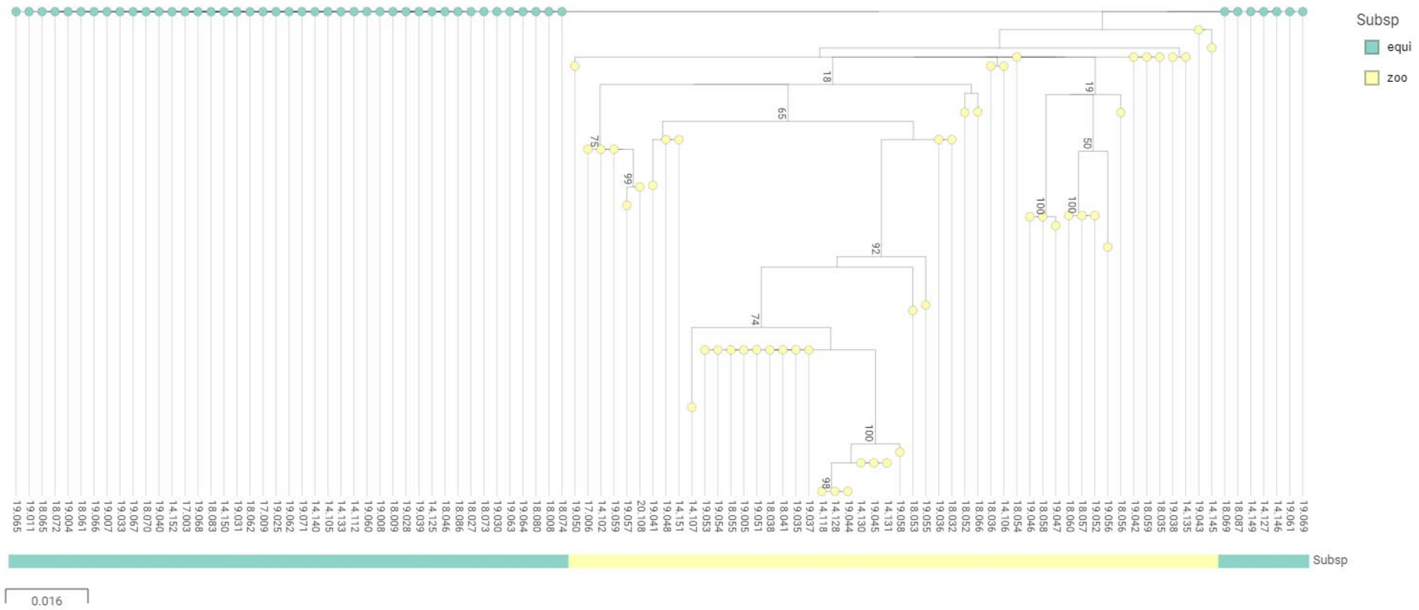

Fig. S2. Phylogenetic tree for SEE (n = 50) and respiratory SEZ (n = 50). Phylogenetic comparisons demonstrate the separation of the isolates by the respective subspecies. The blue squared denote the SEE isolates, and the yellow squares denote the SEZ isolates.

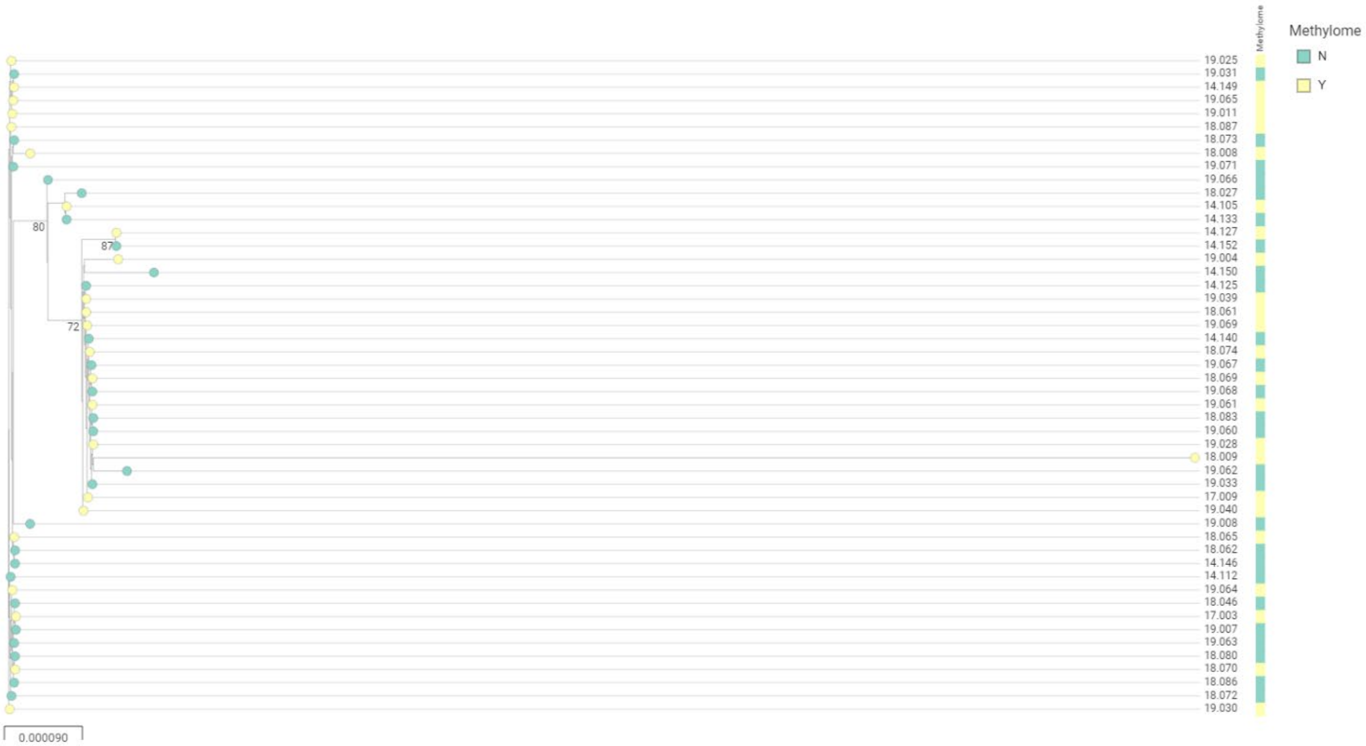

Fig. S3. Phylogenetic tree of SEE (n = 50) isolates. A subset of SEE isolates (n = 24) selected for the methylome analysis based on relatedness in the phylogenetic tree. The blue squares denote SEE isolates that were not (N) used in the methylation analysis, whereas the yellow squares denote SEE isolates that were (Y) used in the methylation analysis.

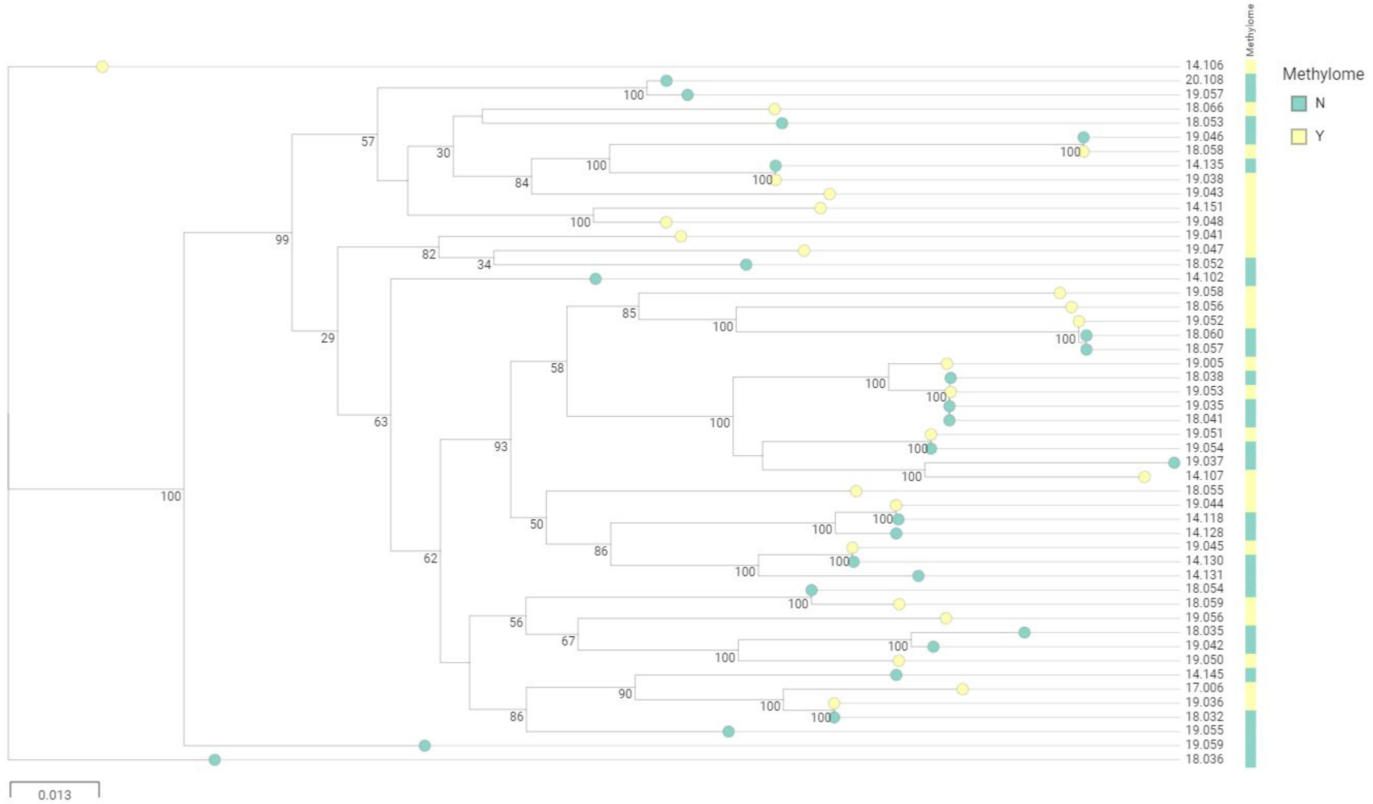

Fig. S4. Phylogenetic tree of SEZ (n = 50) isolates. A subset of SEZ isolates (n = 24) selected for the methylome analysis based on relatedness in the phylogenetic tree. The blue squares denote SEZ isolates that were not (N) used in the methylation analysis, whereas the yellow squares denote SEZ isolates that were (Y) used in the methylation analysis.

#Appendix. Linux and R code used for accessory genome, and methylome analysis.

```
### Streptococcus equi de novo genome assembly with CANU (v1.7) in Linux ###

module load Canu/1.7-intel-2017A-Perl-5.24.0

# command to run pipeline with -pacbio-raw option

canu useGrid=false -p SE_14.105 -d SE_14.105_CANU1.7_out genomeSize=2.1m \

-pacbio-raw ./Duke_Strep_PacBio/FastaFiles/SE_14.105.fasta \

corMhapSensitivity=high corMinCoverage=0 corOutCoverage=100


## Genomes assembled from CANU were annotated using RASTtk
(https://rast.nmpdr.org/rast.cgi)


### SEE (n = 50) and SEZ (n = 50) - Spine, AGEnt, and ClustAGE in Linux ###

#Annotated genomes were reformated using Genbank Reformat
(http://vfsmspineagent.fsm.northwestern.edu/cgi-bin/gbk_reformat.cgi)


#Defining the core genome - Spine

module load Spine/0.3.2-GCCcore-7.3.0-Perl-5.28.0

spine.pl -f genome_files.txt


## Example of text in genome_files.txt below

./SZ_SE_AccessoryGenome/RastAnnotatedGenomes/SZ_14.102.gbk  SZ_14.102   gbk

./SZ_SE_AccessoryGenome/RastAnnotatedGenomes/SZ_14.106.gbk  SZ_14.106   gbk

./SZ_SE_AccessoryGenome/RastAnnotatedGenomes/SE_14.105.gbk  SE_14.105   gbk

./SZ_SE_AccessoryGenome/RastAnnotatedGenomes/SE_14.112.gbk  SE_14.112   gbk

./SZ_SE_AccessoryGenome/RastAnnotatedGenomes/SE_14.125.gbk  SE_14.125   gbk


#Defining the accessory genome - AGEnt

module load AGEnt/0.3.1-GCCcore-7.3.0-Perl-5.28.0

AGEnt.pl -r output.backbone.fasta -q
./SZ_SE_AccessoryGenome/RastAnnotatedGenomes/SE_14.105.gbk -o SE_14.105
##each isolate is run individually


#Clustering and binning the accessory genome elements - ClustAGE

module load Magic-BLAST/1.3.0-x64-linux

module load ClustAGE/0.8-foss-2018b-Perl-5.28.0
```

```
ClustAGE.pl -f age_files.txt --annot annot_files.txt

## Example of text in age_files.txt below
SE_14.105.SE_14.105.accessory.fasta SE_14.105   1
SE_14.112.SE_14.112.accessory.fasta SE_14.112   1
SE_14.125.SE_14.125.accessory.fasta SE_14.125   1
SZ_14.102.SZ_14.102.accessory.fasta SZ_14.102   2
SZ_14.106.SZ_14.106.accessory.fasta SZ_14.106   2
SZ_14.107.SZ_14.107.accessory.fasta SZ_14.107   2

## Example of text in annot_files.txt below
SE_14.105.SE_14.105.accessory_loci.txt    SE_14.105
SE_14.112.SE_14.112.accessory_loci.txt    SE_14.112
SE_14.125.SE_14.125.accessory_loci.txt    SE_14.125
SZ_14.102.SZ_14.102.accessory_loci.txt    SZ_14.102
SZ_14.106.SZ_14.106.accessory_loci.txt    SZ_14.106
SZ_14.107.SZ_14.107.accessory_loci.txt    SZ_14.107

### R code for Accessory Genome Output - SEE and SEZ isolates ###
##R Version 4.0.3
subelem <- read.csv("./ClustAGEOutput_100Genomes/out_subelements.csv", header
= T)
rownames(subelem) <- subelem[,1]
subelem.sums <- subelem[,3:ncol(subelem)]
sums <- colSums(subelem.sums)
#Adding up the bins of accessory genome elements [AGE](1 indicates presence
of AGE, 0 indicates absence)

#Splitting isolates that are SEE
SE.subset <- subelem.sums[1:50,] ## numbers for SEE isolates
SE.sums <- colSums(SE.subset) # sums for only SEE isolates
SE.AGE <- SE.sums[SE.sums == 50]  # keeping bins that == 50
foo <- names(SE.AGE); SE.overall.subset <- subelem.sums[foo] #pulling out
bins that == 50 from combined data
```

```
#Adding up the bins of accessory genome elements [AGE](1 indicates presence of AGE, 0 indicates absence)

SE.overall.sums <- colSums(SE.overall.subset)

SE.overall.sums <- SE.overall.sums[SE.overall.sums == 50] ## from combined data only keeping sites that == 50

names(SE.overall.sums) #Viewing if any sites fit criteria

write.csv(names(SE.overall.sums), "./ClustAGEOutput_100Genomes/50SE.AGE.csv") ##putting the finale output into a CSV file


#Splitting isolates that are SEZ

SZ.subset <- subelem.sums[51:100,] ## numbers for SEZ isolates

SZ.sums <- colSums(SZ.subset) # sums for only SEE isolates

SZ.AGE <- SZ.sums[SZ.sums == 50] # keeping bins that == 50

foo <- names(SZ.AGE); SZ.overall.subset <- subelem.sums[foo]  #pulling out bins that == 50 from combined data

#Adding up the bins of accessory genome elements [AGE](1 indicates presence of AGE, 0 indicates absence)

SZ.overall.sums <- colSums(SZ.overall.subset)

SZ.overall.sums <- SZ.overall.sums[SZ.overall.sums == 50] ## from combined data only keeping sites that == 50

names(SZ.overall.sums) #Viewing if any sites fit criteria

write.csv(names(SZ.overall.sums), "./ClustAGEOutput_100Genomes/50SZ.AGE.csv") ##putting the finale output into a CSV file


## Keeping the AGE that only have 95% of the protein identified in the AGE analysis

## Prior to being brought back into the R the CSV files were modified to put each individual protein into its own row using Notepad++

SEE.prot <- read.csv("./ClustAGEOutput_100Genomes/AGEs_Proteins_SEE.csv", header = T)

SEZ.prot <- read.csv("./ClustAGEOutput_100Genomes/AGEs_Proteins_SEZ.csv", header = T)


library(dplyr); packageVersion("dplyr") ##1.0.2

#Filtering the protein list

SEE.prot.95 <- SEE.prot %>% filter(Percent >= 95.00)

SEZ.prot.95 <- SEZ.prot %>% filter(Percent >= 95.00)
```

```
#Writing the outputs to a comma separated file

write.csv(SEE.prot.95,
"./ClustAGEOutput_100Genomes/AGEs_Proteins_SEE_95.csv", quote = F)

write.csv(SEZ.prot.95,
"./ClustAGEOutput_100Genomes/AGEs_Proteins_SEZ_95.csv", quote = F)



###############################################################################

### BaseMod Methylation pipeline for SEE (n = 24) & SEZ (n = 24) isolates using the SMRT-Link 8 command line tools - Linux ###

## Example of pipeline for individual isolate


module load SMRT-Link/8.0.0.80529-cli-tools-only


#Aligning the raw BAM reads to the reference

pbmm2 align SE_4047.fasta SE_14.149.bam SE_14.149.aligned.bam

## pbmm2 align SZ_H70.fasta SZ_14.151.bam SZ_14.151.aligned.bam ## alignment for SEZ isolates

#Creating an index for the reference and the Streptococcus equi isolates

samtools faidx SE_4047.fasta ### Indexing the SEE 4047 reference gennome

#samtools faidx SZ_H70.fasta ### Indexing the SEZ H70 reference genome

pbindex SE_14.149.aligned.bam

#Analyzing the aligned sequences for base modifcations

ipdSummary SE_14.149.aligned.bam --reference SE_4047.fasta --gff SE_14.149.basemods.gff --csv SE_14.149.basemods.csv --pvalue 0.001 --numWorkers 16 --identify m4C,m6A

#Identifying any consensus motifs

motifMaker find -f SE_4047.fasta -g SE_14.149.basemods.gff -o SE_14.149.motifs.csv ### requires more computational sources than the ipdSummary command

#Creating a GFF file with all of the modification that are part of the motifs

motifMaker reprocess -f SE_4047.fasta -g SE_14.149.basemods.gff -m SE_14.149.motifs.csv -o SE_14.149.motifs.gff


### R code for to filter BaseMod GFF files prior to whole genome comparison set with BEDTools ###

##R Version 4.0.3
```

```
library(ape); packageVersion("ape") ## ape: 5.4.1

SE.14.127 <- read.gff("./Strep_equi/Motif_Gff/SE_14.127.motifs.gff", GFF3 =
TRUE)

##Example of code for a single isolate


library(dplyr); packageVersion("dplyr") ##1.0.3

library(tidyr); packageVersion("tidyr") ##1.1.2


##### S. equi 14-127 #####

SE.14.127_filtered <- filter(SE.14.127, !grepl('modified_base', type))
#removing instances of modified base

SE.14.127_filt.motif <- filter(SE.14.127_filtered, grepl('motif',
attributes)) #pulling out modification with motifs

out <- strsplit(as.character(SE.14.127_filt.motif$attributes), ";");

SE.14.127_filt.motif_attributes <- data.frame(t(sapply(out, '[')));

colnames(SE.14.127_filt.motif_attributes) <- c("context", "motif",
"coverage", "IPDRatio", "id", "identifcationQv") #splitting the attributes
column into new columns by semi-colon

SE.14.127_filt.motif <- cbind(SE.14.127_filt.motif,
SE.14.127_filt.motif_attributes)


SE.14.127_filt.nomotif <- filter(SE.14.127_filtered, !grepl('motif',
attributes)) # pulling out modifications with out motifs

out <- strsplit(as.character(SE.14.127_filt.nomotif$attributes), ";");
SE.14.127_filt.nomotif_attributes <- data.frame(t(sapply(out, '[')));
colnames(SE.14.127_filt.nomotif_attributes) <- c("coverage", "context",
"IPDRatio", "identifcationQv")

SE.14.127_filt.nomotif <- cbind(SE.14.127_filt.nomotif,
SE.14.127_filt.nomotif_attributes) #splitting the attributes column into new
columns by semi-colon

na <- rep(NA, nrow(SE.14.127_filt.nomotif)); SE.14.127_filt.nomotif$motif <-
na ; SE.14.127_filt.nomotif$id <- na ##creating columns of NAs to match
columns seen in data with motifs


#Combining the data with and without motifs

SE.14.127_filtered <- rbind(SE.14.127_filt.motif, SE.14.127_filt.nomotif)

out <- strsplit(as.character(SE.14.127_filtered$identifcationQv), "=");
SE.14.127_Qv <- data.frame(t(sapply(out, '[')));
```

```
colnames(SE.14.127_Qv) <- c("Qv", "QvScore"); SE.14.127_Qv$QvScore <-
as.numeric(SE.14.127_Qv$QvScore)


SE.14.127_filtered <- cbind(SE.14.127_filtered, SE.14.127_Qv) #pulling out
the QV score values


SE.14.127_QvScore30 <- filter(SE.14.127_filtered, QvScore >= 30) #Keeping
only methylation with a QV score >= 30


#outputting the filtered data in text and gff file formats

write.table(SE.14.127_QvScore30, "./Strep_equi/SE.14.127_filtered.txt", sep =
"\t", quote = F)

library(rtracklayer); packageVersion("rtracklayer") ##1.48.0

export(SE.14.127_QvScore30, "./Strep_equi/SE.14.127_filtered.gff", format =
"gff3")


###Creating a annotated GFF file with the methylation events across all SEE &
SEZ isolates by reference genome in Linux ###

module load BEDTools/2.29.2-GCC-9.3.0


## Code for a SEE isolates

bedtools annotate -i SEE_4047.gff3 -files SE.14.105_filtered.gff
SE.17.003_filtered.gff SE.19.025_filtered.gff SE.14.127_filtered.gff \

SE.17.009_filtered.gff SE.18.008_filtered.gff SE.18.061_filtered.gff
SE.18.074_filtered.gff SE.18.087_filtered.gff SE.19.039_filtered.gff \

SE.19.040_filtered.gff SE.19.061_filtered.gff SE.19.065_filtered.gff
SE_14.149_filtered.gff SE_18.009_filtered.gff SE_18.065_filtered.gff \

SE_18.069_filtered.gff SE_18.070_filtered.gff SE_19.004_filtered.gff
SE_19.011_filtered.gff SE_19.028_filtered.gff SE_19.064_filtered.gff \

SE_19.069_filtered.gff SE_19.030_filtered.gff > All_SEE_Methylation_24.gff

## Code for SEZ isolates

bedtools annotate -i SZ_H70.gff3 -files SZ.17.006_filtered.gff
SZ.19.005_filtered.gff SZ.14.151_filtered.gff SZ.18.055_filtered.gff \

SZ.18.058_filtered.gff SZ.18.059_filtered.gff SZ.18.066_filtered.gff
SZ.19.045_filtered.gff SZ.19.058_filtered.gff SZ.19.038_filtered.gff \

SZ.19.043_filtered.gff SZ.19.052_filtered.gff SZ.19.036_filtered.gff
SZ.14.106_filtered.gff SZ.14.107_filtered.gff SZ.18.056_filtered.gff \

SZ.19.041_filtered.gff SZ.19.044_filtered.gff SZ.19.047_filtered.gff
SZ.19.048_filtered.gff SZ.19.050_filtered.gff SZ.19.051_filtered.gff \
```

```
SZ.19.053_filtered.gff SZ.19.056_filtered.gff > All_SEZ_Methylation_24.gff


### R code for identify site of methylation in SEE & SEZ isolates on homologous proteins (separately) ###

AllSEE_methy_anno <- read.delim("./All_SEE_Methylation_24_edited.txt", header=FALSE)

AllSEZ_methy_anno <- read.delim("./All_SEZ_Methylation_24_edited.txt", header=FALSE)


methy.localSEE <- AllSEE_methy_anno[,7:ncol(AllSEE_methy_anno)]

se <- c("SE.14.105", "SE.17.003", "SE.19.025", "SE.14.127", "SE.17.009", "SE.18.008", "SE.18.061", "SE.18.074",

        "SE.18.087", "SE.19.039", "SE.19.040", "SE.19.061", "SE.19.065", "SE_14.149", "SE_18.009", "SE_18.065", "SE_18.069",

        "SE_18.070", "SE_19.004", "SE_19.011", "SE_19.028", "SE_19.064", "SE_19.069", "SE_19.030")

colnames(methy.localSEE) <- se


methy.localSEZ <- AllSEZ_methy_anno[,7:ncol(AllSEZ_methy_anno)]

sez <- c("SZ.17.006", "SZ.19.005", "SZ.14.151", "SZ.18.055", "SZ.18.058", "SZ.18.059", "SZ.18.066", "SZ.19.045", "SZ.19.058",

         "SZ.19.038", "SZ.19.043", "SZ.19.052", "SZ.19.036", "SZ.14.106", "SZ.14.107", "SZ.18.056", "SZ.19.041", "SZ.19.044",

         "SZ.19.047", "SZ.19.048", "SZ.19.050", "SZ.19.051", "SZ.19.053", "SZ.19.056")

colnames(methy.localSEZ) <- sez


### Keeping rows with only zeros in SEE isolates

NO.methy.localSEE <- methy.localSEE[apply(methy.localSEE[,-1], 1, function(x) all(x==0)),]

NO.methy.localSEE <- NO.methy.localSEE[rowSums(NO.methy.localSEE) == 0,]

B <- row.names(NO.methy.localSEE)

SEE_No_MethyAnnotate_Subset <- AllSEE_methy_anno[B, ]

dim(SEE_No_MethyAnnotate_Subset)


### Keeping rows with only zeros in SEZ isolates

NO.methy.localSEZ <- methy.localSEZ[apply(methy.localSEZ[,-1], 1, function(x) all(x==0)),] ### Keeping rows with only zeros
```

```
NO.methy.localSEZ <- NO.methy.localSEZ[rowSums(NO.methy.localSEZ) == 0,]

B <- row.names(NO.methy.localSEZ)

SEZ_No_MethyAnnotate_Subset <- AllSEZ_methy_anno[B, ]

dim(SEZ_No_MethyAnnotate_Subset)


## Removing ANY rows that contain a zero value in SEE isolates

ALL.methy.localSEE <- methy.localSEE[apply(methy.localSEE, 1,function(x)
!any(x==0)),]

B <- row.names(ALL.methy.localSEE)

SEE_ALL_MethyAnnotate_Subset <- AllSEE_methy_anno[B, ]

dim(SEE_ALL_MethyAnnotate_Subset)


## Removing ANY rows that contain a zero value in SEZ isolates

ALL.methy.localSEZ <- methy.localSEZ[apply(methy.localSEZ, 1,function(x)
!any(x==0)),]

B <- row.names(ALL.methy.localSEZ)

SEZ_ALL_MethyAnnotate_Subset <- AllSEZ_methy_anno[B, ]

dim(SEZ_ALL_MethyAnnotate_Subset)


prot.list <- read.delim("SEEvsSEZ.txt", header = T)

names(prot.list)


library(dplyr)

############# Using PATRIC output with H70 as ref compared to 4047 & ATCC
39506 ###############

SEZ.prot.list <-
read.delim("./SEEvsSEZ_ProteinList/genome_comparison_H70_Ref_edited.txt",
header = T)

names(SEZ.prot.list)


SEZ.prot.list <- filter(SEZ.prot.list, SEE_4047_percent_identity >= 0.99 &
SEE_4047_seq_coverage >= 0.99)

H70.filt_prot.list <- filter(SEZ.prot.list, SEE_ATCC39506_percent_identity >=
0.99 & SEE_ATCC39506_seq_coverage >= 0.99)

nrow(H70.filt_prot.list)

## 623
```

```
# Merging the filtered genes that are >= 99% to entire genome comparison list
from patric

SZH70.entirelist <-
read.delim("./SEEvsSEZ_ProteinList/genome_comparison_H70_Ref.txt", header =
T)

merged.H70 <- SZH70.entirelist[SZH70.entirelist$ref_SEZH70_genome_gene %in%
H70.filt_prot.list$H70_ref_genome_gene,]


locus.tag_4047 <- merged.H70$SEE_4047_locus_tag

locus.tag_H70 <- merged.H70$ref_SEZH70_genome_locus_tag

locus.tag <- data.frame(cbind(locus.tag_H70, locus.tag_4047))

write.table(locus.tag, "./SEEvsSEZ_ProteinList/Combined_LocusTags.txt", sep =
"\t", quote = F)


## Reading into R the annotated presence and absence methylation data from
SEE & SEZ

SEE_NoMeth_LocusTag <-
read.delim("./SecondSet/SEE_No_MethyAnnotate_Subset.txt", header = T)

SEZ_NoMethy_LocusTag <-
read.delim("./SecondSet/SEZ_No_MethyAnnotate_Subset.txt", header = T)

SEE_ALLMethy_LocusTag <-
read.delim("./SecondSet/SEE_ALL_MethyAnnotate_Subset.txt", header =T)

SEZ_ALLMethy_LocusTag <-
read.delim("./SecondSet/SEZ_ALL_MethyAnnotate_Subset.txt", header = T)


## Adding the protein IDs to the methylation presence and absence data

SEE_LocusTargets_NoMethy <- SEE_NoMeth_LocusTag[SEE_NoMeth_LocusTag$V32 %in%
locus.tag$locus.tag_4047,]

SEZ_LocusTargets_NoMethy <- SEZ_NoMethy_LocusTag[SEZ_NoMethy_LocusTag$V32
%in% locus.tag$locus.tag_H70,]

SEE_LocusTargets_ALLMethy <- SEE_ALLMethy_LocusTag[SEE_ALLMethy_LocusTag$V32
%in% locus.tag$locus.tag_4047,]

SEZ_LocusTargets_ALLMethy <- SEZ_ALLMethy_LocusTag[SEZ_ALLMethy_LocusTag$V32
%in% locus.tag$locus.tag_H70,]


### Adding a column name to the last column of each dataframe so they can be
merged

colnames(SEE_LocusTargets_NoMethy)[ncol(SEE_LocusTargets_NoMethy)] <-
"locus.tag_4047"
```

```
colnames(SEZ_LocusTargets_NoMethy)[ncol(SEZ_LocusTargets_NoMethy)] <- 
"locus.tag_H70"

colnames(SEE_LocusTargets_ALLMethy)[ncol(SEE_LocusTargets_ALLMethy)] <- 
"locus.tag_4047"

colnames(SEZ_LocusTargets_ALLMethy)[ncol(SEZ_LocusTargets_ALLMethy)] <- 
"locus.tag_H70"


### Adding the homologous SEE/SEZ protien to the methylation profile.

library(dplyr); packageVersion("dplyr") ##1.0.3

SEE_LocusTargets_NoMethy <- full_join(SEE_LocusTargets_NoMethy, locus.tag, by 
= "locus.tag_4047")

SEE_LocusTargets_NoMethy <- na.omit(SEE_LocusTargets_NoMethy)

dim(SEE_LocusTargets_NoMethy)

# [1] 1376   34


SEZ_LocusTargets_NoMethy <- full_join(SEZ_LocusTargets_NoMethy, locus.tag, by 
= "locus.tag_H70")

SEZ_LocusTargets_NoMethy <- na.omit(SEZ_LocusTargets_NoMethy)

dim(SEZ_LocusTargets_NoMethy)

#[1] 484   34


SEE_LocusTargets_ALLMethy <- full_join(SEE_LocusTargets_ALLMethy, locus.tag, 
by = "locus.tag_4047")

SEE_LocusTargets_ALLMethy <-na.omit(SEE_LocusTargets_ALLMethy)

dim(SEE_LocusTargets_ALLMethy)

#[1] 251  34


SEZ_LocusTargets_ALLMethy <- full_join(SEZ_LocusTargets_ALLMethy, locus.tag, 
by = "locus.tag_H70")

SEZ_LocusTargets_ALLMethy <-na.omit(SEZ_LocusTargets_ALLMethy)

dim(SEZ_LocusTargets_ALLMethy)

#[1] 28  34


library(plyr); packageVersion("plyr") ###‘1.8.6’

### Combining the absence of SEE methylation locations with the SEZ presence 
data at homologous proteins
```

```
SEE.No_vs_SEZ.All <-
SEE_LocusTargets_NoMethy[SEE_LocusTargets_NoMethy$locus.tag_H70 %in%
SEZ_LocusTargets_ALLMethy$locus.tag_H70,]

dim(SEE.No_vs_SEZ.All)

## [1] 34 34

count(SEE.No_vs_SEZ.All$locus.tag_4047)

write.table(SEE.No_vs_SEZ.All, 'SEE.No_vs_SEZ.All_04Jan21_48isolates.txt',
sep = "\t", quote = F)


### Combining the absence of SEZ methylation locations with the SEE presence
data at homologous proteins

SEE.All_vs_SEZ.No <-
SEE_LocusTargets_ALLMethy[SEE_LocusTargets_ALLMethy$locus.tag_H70 %in%
SEZ_LocusTargets_NoMethy$locus.tag_H70,]

dim(SEE.All_vs_SEZ.No)

## [1] 117  34

count(SEE.All_vs_SEZ.No$locus.tag_4047)

write.table(SEE.All_vs_SEZ.No, 'SEE.All_vs_SEZ.No_04Jan21_48isolates.txt',
sep = "\t", quote = F)




#### Checking to be sure sites at which methylation occurred in all SEE
isolates is homogenous in methylation type and location.

library(dplyr); packageVersion("dplyr") ##1.0.2

SE.list <-
list(SE.14.105,SE.14.127,SE.14.149,SE.17.003,SE.17.009,SE.18.008,SE.18.009,
SE.18.061,SE.18.065,SE.18.069,SE.18.070,SE.18.074,SE.18.087,SE.19.004,
SE.19.011,SE.19.025,SE.19.028,SE.19.030,SE.19.039,SE.19.040,SE.19.061,
SE.19.064,SE.19.065,SE.19.069)

##Example of for a single homologous protein

SE.SEQ_0045 <- lapply(SE.list, function(x) subset(x, x$start >= 56643 &
x$start <= 57695));

SE.SEQ_0045 <- bind_rows(SE.SEQ_0045) #selecting methylation that occurred on
SEQ_0045


library(plyr); packageVersion("plyr") ##1.8.6

count(SE.SEQ_0045$start)


library(dplyr); packageVersion("dplyr") ##1.0.2
```

```
#Subsetting the dataframe by sites were all 24 SEE genomes have methylation
present

All.SE.SEQ_0045 <- subset(SE.SEQ_0045, SE.SEQ_0045$start == 56855)


## Checking the time of methylation that occurs at those locations

count(All.SE.SEQ_0045$type)
```
